# Sex differences in cancer risk among germline p53 mutation carriers

**DOI:** 10.1038/sj.bjc.6602032

**Published:** 2004-07-27

**Authors:** A Chompret, L Brugières, C Bonaïti-Pellié, J Feunteun

**Affiliations:** 1 Consultation de Génétique Institut Gustave-Roussy, Villejuif, France; 2 Département de Pédiatrie Institut Gustave-Roussy, Villejuif, France; 3 INSERM U535 ‘Génétique épidémiologique et structure des populations humaines’, Hôpital Paul Brousse, Villejuif, France; 4 Laboratoire de Génétique Oncologique, UMR 8125, Institut Gustave-Roussy Villejuif, France; 5 Hematology/Oncology Division, General Hospital of Annemasse, BP 525, Annemasse Cedex 74107, France

**Sir,**

In a recent issue of the *American Journal of Human Genetics*, Hwang *et al* (2003) reported sex difference in cancer risks among p53 germline mutation carriers from a follow-up study of 56 carriers belonging to seven families from a screening of 107 kindreds ascertained through patients with childhood soft tissue sarcoma. We wish to point out that we have reported such sex difference 3 years ago (Chompret *et al*, 2000). The satisfaction for reading a report confirmatory of our work is mixed by the lack of acknowledgment and discussion of our contribution.

Our estimation of cancer risk was based on family data of 295 children treated for a solid tumour at the Institut Gustave-Roussy in Villejuif (France), and screened for p53 germline mutations. This sample was selected among 2691 children treated for a solid tumour, whose family history was investigated between 1991 and 1997 through either of the following criteria: (1) at least one first- or second-degree relative affected by early-onset cancer (45 years or younger); (2) multiple primary cancers in the proband regardless of family history. A total of 17 mutations were found, four of which were *de novo* mutations, and the remaining 13 were segregating in the families. Blood samples were obtained from first-degree relatives and, when a relative (first- or second-degree or first cousin) was affected by early-onset cancer (i.e. diagnosed before age 46), from all the available family members in that branch of the pedigree. An original method of estimation of cancer risk in carriers (Le Bihan *et al*, 1995) that takes into account the particular ascertainment design of this family sample was used. Cumulative cancer risks by ages 16, 45 and 85 years were estimated to be respectively 19, 41 and 73% in males, and 12, 84 and 100% in females. There was no difference between males and females in childhood, with an average risk of 15%, but the risks were found to be significantly higher in females than in males in adulthood, and in particular in the 16–45 age class.

We appreciate that cancer risk estimates, obtained from informative and *a priori* free of any ascertainment bias data, by Hwang *et al* (2003) are quite similar to ours. The cumulative risks estimated by these authors by ages 20, 30, 40 and 50 years were, respectively, 18, 49, 77 and 93% in female carriers, and 10, 21, 33 and 68% in male carriers. The original finding in this study is that the difference in risk between males and females cannot be explained by the sex-specific cancer, since the difference remained after exclusion of breast, ovary and prostate cancer. Furthermore, they were able to evaluate more accurately the risks at various tumor sites, which was not possible from our data. Only relative risks were estimated, although it would have been interesting to estimate the absolute risks of second cancers, a major issue for germline p53 carriers.

The comparison of the results of Hwang *et al* (2003) to our data allows to draw an interesting conclusion on p53 germline mutations. Although our study used far less stringent criteria than those defining the Li–Fraumeni syndrome (Li *et al*, 1988) for selecting families, most of the mutations were found in families with strong familial aggregation, suggesting that low penetrance mutations are probably rare. These findings are confirmed by the study of Hwang *et al* (2003), which, in spite of less stringent criteria, also finds mutations in families with strong familial aggregation: 67 malignant tumours were found in 45 individuals in 56 carriers within only seven families. If low-penetrance mutation was not rare, their estimates of cancer risk would have been lower than ours, which is not the case.

That penetrance is incomplete in men has an important implication on the diagnosis of the syndrome. Indeed, if a p53 mutation segregates in a family in which, by chance, most carriers are males, the classical criteria for diagnosing a Li–Fraumeni syndrome (Li *et al*, 1988) are most unlikely to be reached and looser criteria (Birch *et al*, 1994; Varley *et al*, 1997; Chompret *et al*, 2001) must be preferred.

*Editor's note*: When we contacted Dr Louise C Strong, one of the principle authors on the paper published in the *American Journal of Human Genetics* to which Dr Feunteun referred, Dr Strong kindly replied that she became aware of Dr Feunteun's prior paper shortly after her own paper was published and would certainly have cited it had she been aware of it earlier.
